# *LuxCDABE—*Transformed Constitutively Bioluminescent *Escherichia coli* for Toxicity Screening: Comparison with Naturally Luminous *Vibrio fischeri*

**DOI:** 10.3390/s110807865

**Published:** 2011-08-11

**Authors:** Imbi Kurvet, Angela Ivask, Olesja Bondarenko, Mariliis Sihtmäe, Anne Kahru

**Affiliations:** 1 Laboratory of Molecular Genetics, National Institute of Chemical Physics and Biophysics, Akadeemia tee 23, Tallinn 12618, Estonia; E-Mails: imbi.kurvet@kbfi.ee (I.K.); olesja.bondarenko@kbfi.ee (O.B.); mariliis.sihtmae@kbfi.ee (M.S.); 2 Department of Food Processing, Tallinn University of Technology, Ehitajate tee 5, Tallinn 19086, Estonia; 3 Department of Gene Technology, Tallinn University of Technology, Ehitajate tee 5, Tallinn 19086, Estonia; 4 Department of Chemical Engineering, Tallinn University of Technology, Ehitajate tee 5, Tallinn 19086, Estonia

**Keywords:** bioluminescence, *luxCDABE*, toxicity, heavy metals, anilines, high throughput assay

## Abstract

We show that *in vitro* toxicity assay based on inhibition of the bioluminescence of recombinant *Escherichia coli* encoding thermostable luciferase from *Photorhabdus luminescens* is a versatile alternative to *Vibrio fischeri* Microtox™ test. Performance of two *luxCDABE*-transformed *E. coli* MC1061 constructs (pDNlux) and (pSLlux) otherwise identical, but having 100-fold different background luminescence was compared with the performance of *V. fischeri.* The microplate luminometer and a kinetic Flash-Assay test format was used that differently from Microtox test is also applicable for high throughput analysis. Toxic effects (30-s till 30-min EC_50_) of four heavy metals (Zn, Cd, Hg, Cu) and three organic chemicals (aniline, 3,5-dichloroaniline and 3,5-dichlorophenol) were studied. Both *E. coli* strains had comparable sensitivity and the respective 30-min EC_50_ values highly correlated (log-log R^2^ = 0.99; p < 0.01) showing that the sensitivity of the recombinant bacteria towards chemicals analyzed did not depend on the bioluminescence level of the recombinant cells. The most toxic chemical for all used bacterial strains (*E. coli, V. fischeri*) was mercury whereas the lowest EC_50_ values for Hg (0.04–0.05 mg/L) and highest EC_50_ values for aniline (1,300–1,700 mg/L) were observed for *E. coli* strains. Despite of that, toxicity results obtained with both *E. coli* strains (pSLlux and pDNlux) significantly correlated with *V. fischeri* results (log-log R^2^ = 0.70/0.75; p < 0.05/0.01). The use of amino acids (0.25%) and glucose (0.05%)-supplemented M9 medium instead of leucine-supplemented saline significantly (p < 0.05) reduced the apparent toxicity of heavy metals to both *E. coli* strains up to three orders of magnitude, but had little or no complexing effect on organic compounds. Thus, *P. luminescens luxCDABE*-transformed *E. coli* strains can be successfully used for the acute toxicity screening of various types of organic chemicals and heavy metals and can replace *V. fischeri* in certain cases where the thermostability of luciferase >30 °C is crucial. The kinetic Flash Assay test format of the bioluminescence inhibition assay facilitates high throughput analysis. The assay medium, especially in case of testing heavy metals should be a compromise: optimal for the viability/luminescence of the recombinant test strain and of minimum complexing potential.

## 1. Introduction

Over the last twenty five years, alternative, non-animal test systems (mainly eukaryotic cell cultures) have been introduced to supplement and, in some cases, to replace toxicity tests using animals [1], contributing to the 3R’s concept (Replacement, Reduction, Refinement of test animals) [2]. For initial toxicity screening of chemicals, bacteria are an additional attractive alternative to eukaryotic organisms. The most well-known bacterial *in vitro* test is Ames assay with *Salmonella typhimurium* [3], which may predict genotoxic effects of chemicals also to higher organisms (e.g., humans). One of the most widely used bacterial *in vitro* assays is also the Microtox™ test, which uses the inhibition of bioluminescence of *Vibrio fischeri* NRRL-B-11177 as a toxicity endpoint [4]. *V. fischeri* are naturally luminescent Gram-negative marine bacteria also known as *Photobacterium phosphoreum* NRRL-B-11177 [5] and/or *Aliivibrio fischeri* [6] in which *luxCDABE* genes are responsible for their bioluminescent reaction. When strongly expressed, a single bacterium may emit 10^4^ or 10^5^ photons s^−1^ [7]. *LuxCDE* genes encode a fatty acid reductase complex involved in synthesis of the long chain aliphatic aldehyde (RCHO) substrate for the luminescence reaction catalyzed by the luciferase LuxAB subunits [8]. Bacterial luciferase enzymes mediate the oxidation of reduced flavin mononucleotide (FMNH_2_) and RCHO by molecular oxygen (O_2_) to produce bioluminescence (blue-green light emission) with a maximum intensity at about 490 nm. The overall reaction can be summarized as:

(1) $$\mathrm{FMN}H_{2}+\mathrm{RCHO}+O_{2}\to \mathrm{FMN}+H_{2}O+\mathrm{RCOOH}+\mathrm{light}$$

For the regeneration of FMNH_2_ cellular NADH_2_ is needed and due to that the bioluminescence of the bacteria is intrinsically tied to their central metabolism. Thus, any damage of cellular metabolism caused by the toxicity of a sample could be monitored by measuring the change of light output of bacteria, the degree of toxicity being proportional to the light loss [4,9]. The *V. fischeri* bioluminescence inhibition assay has proven as a rapid, simple and sensitive method in toxicity testing of a wide spectrum of chemicals [10–13] and environmental samples including wastewater, solid waste, soil and sludge extracts [14–17]. A number of comparisons of the *V. fischeri* test (Microtox™) with different ecotoxicological assays [18] but also with various other types of toxicity assays has been made. Thus, analysis of the toxicity data for 47 MEIC reference chemicals showed that the EC_50_ values of *P. phosphoreum* bioluminescence inhibition assay correlated with literature data on acute toxicity data for daphnids, fish, animal and human cell lines, rodents, dog and man whereas the log-log correlation coefficients (R^2^) ranged between 0.20–0.79, depending on the data compared [19]. Thus, the naturally luminescent bacteria have already proven their potential in toxicity testing. Kinetic format of the *V. fischeri* test—a Flash-Assay—has been recently standardized [20] and is applicable for the analysis of sediments, solid wastes, colored samples [21] as well as for synthetic nanoparticles [13].

However, the use of *V. fischeri* in toxicity testing has also some limitations: due to the marine origin of bacteria, its application for freshwater and terrestrial toxicity analysis, but also for *in vitro* toxicity screening may raise questions. The temperature suggested for Microtox assay (+15 °C) is not compatible with conventional plate luminometers as well as needed high salinity (2% NaCl) is not suitable for mimicking certain environments. Also, for the creation of recombinant luminous constructs with temperature optimum at >30 °C, *V. fischeri* luciferase is not suitable due to lack of thermostability at these temperatures [22]. Due to that, luciferase encoded from *luxCDABE* genes from *Photorhabdus luminescens* has been often used for creation of recombinant luminescent bioreporters [22–25]. *P. luminescens* was isolated from a human wound and had the optimum temperature for growth and bioluminescence at 33 °C whereas the optimal temperature for the activity of purified luciferase was 40 °C [26]. Theoretically, the recombinant bacteria encoding thermostable *luxCDABE* could be a good alternative (broad temperature range, robustness) to conventional *V. fischeri* in toxicity screening applications. *Prior* to that, the performance of new recombinant bacteria with thermostable *luxCDABE* genes should be carefully evaluated in comparison to *V. fischeri*. Once validated, this test system could be also relevant for the discovering of group-, genus- or strain-specific antibacterial compounds. Some such systems have been developed and applied for (i) non-invasive *in vivo* bioluminescence imaging of virulent *Staphylococcus aureus* [27], *Mycobacterium tuberculosis* and *M. smegmatis* [28] and (ii) for studying antimicrobial efficiency of cationic peptides [29] and rhamnolipid-surfactants [30] against *Pseudomonas aeruginosa*.

For the further developing of such kind of models for the toxicity analysis, it is important to be sure that the sensitivity of bacteria to chemicals is attributed to the host bacterium and the test conditions and is not affected by the luminescent system used. Indeed, the bioluminescence is metabolically costly being responsible for 12% (*Vibrio harveyi*) and 20% (*V. fischeri*) of the total cellular energy requirement [31], which may theoretically compromise cellular resistance mechanisms and lead to the differences in sensitivity in bacteria with different background luminescence.

The main aim of the current study was to demonstrate that thermostable *luxCDABE*-transformed constitutively luminescent *E. coli* can be used as an alternative to *V. fischeri* for evaluation of acute toxicity of chemicals. Constitutively luminescent *E. coli* could be a suitable model for hygienic and medical microbiology for the discovering of the most effective substance against the virulent *E. coli* strains. We compared the toxicities of four heavy metals (Zn, Cu, Cd, Hg) and three organic chemicals (aniline, 3,5-dichloroaniline and 3,5-dichlorophenol) to two artificially bioluminescent *E. coli* MC1061 strains with 100-fold different background bioluminescence and naturally luminous *V. fischeri.* All the chosen test chemicals are important environmental toxicants and often used as biocides but differ in their modes of toxic action. To optimize the test conditions, two test media of different composition and complexing potential and exposure times of 30-s, 15-min and 30-min were used. Flash-Assay format of the bioluminescence inhibition assay in the microplate luminometer was used throughout.

## 2. Materials and Methods

### 2.1. Chemicals

All standard chemicals used were >99% of purity: ZnSO_4_·7H_2_O and CuSO_4_ were purchased from Alfa-Aesar (Karlsruhe, Germany); HgCl_2_, CdCl_2_·H_2_O and 3,5-dichlorophenol from Riedel-de-Haën Seelze, Germany). Aniline and 3,5-dichloroaniline were purchased from Sigma-Aldrich (Steinheim, Germany). The following stock solutions of standard chemicals: 27.2 mg/mL for HgCl_2_, 287.0 mg/mL for ZnSO_4_·7H_2_O, 159.5 mg/mL for CuSO_4_, 201 mg/mL for CdCl_2_·H_2_O, 8.0 mg/mL for aniline, 2.6 mg/mL for 3,5-dichlorophenol, 0.2 mg/mL for dichloroaniline were prepared in MilliQ water and stored in the dark. Before testing, the stock-solutions were diluted in MilliQ water (for *E. coli* tests) or in 2% NaCl (for *V. fischeri* tests).

### 2.2. Luminescent Bacterial Strains and their Preparation for Toxicity Testing

#### 2.2.1. *Escherichia coli* Strains

Constitutively bioluminescent *E. coli* MC1061(pDNlux) and *E. coli* MC1061(pSLlux) constructed earlier by Ivask *et al*. [25] (Table 1) were used for toxicity testing. Bacteria were maintained in LB agar medium (LabM, Lancashire, UK) [32] supplemented with appropriate antibiotics (Table 1). For the toxicity tests, bacteria were cultivated (on a shaker at 200 rpm, 30 °C) overnight in 3 mL of M9 medium containing (per l): 6 g of Na_2_HPO_4_, 3 g of KH_2_PO_4_, 0.5 g of NaCl, 1 g of NH_4_Cl, 0.25 g of MgSO_4_·7H_2_O, 0.01 g of CaCl_2_ supplemented with glucose (Cerastar, Denmark) (final concentration 0.1%), acid hydrolysate of casein (cas-AA; LabM, Lancashire, UK) (final concentration 0.5%) and appropriate antibiotics (Table 1). 1 mL of overnight culture of *E. coli* was added to 50 mL of fresh medium and grown till logarithmic growth phase OD_600_ = 0.6.

The mid-exponential phase culture was further: (i) diluted until OD_600_ of 0.1 with M9 medium supplemented with 0.1% glucose and 0.5% cas-AA (further referred to as “GAA-M9”) or (ii) centrifuged at 5,000 rpm for 5 min and washed twice with 0.9% NaCl supplemented with 0.01% leucine (Sigma-Aldrich, Steinheim, Germany) (further referred to as “Leu-saline”) and then diluted with the same medium till OD_600_ of 0.1. As in the toxicity test bacterial suspension is added to equal volume of chemical dilution prepared in water, the final composition of test media for *E. coli* is as follows: GAA-M9 (0.05% glucose, 0.25% casAA, 50% M9) and Leu-saline (0.005% leucine and 0.45% NaCl). Leucine is added due to the auxotrophy of this *E. coli* strain to leucine (Table 1) and 0.45% NaCl was optimal for luminescence of recombinant *Escherichia coli* strain K12 TG1, carrying *luxCDABE* genes of *Photobacterium leiognathi* 54D10 as shown by Deryabin and Aleshina [35]. For *V. fischeri* (Microtox™ standard operational procedure) as well as for *P. phosphoreum* KCTC 2852 [36] the optimal is 2% NaCl which was used also in the current study. Bacterial suspensions prepared in GAA-M9 were used for toxicity test immediately but bacterial suspensions in Leu-saline were incubated for 1.5 h at room temperature to stabilize the luminescence. The number of bacterial cells in the test was determined by counting the number of bacterial colony forming units (CFU) before the test on agarized LB media supplemented with appropriate antibiotics (Table 1). The number of *E. coli* cells in the test was 3.2 ± 0.63 × 10^7^ per mL.

#### 2.2.2. *Vibrio fischeri* Strains

*Vibrio fischeri* NRRL-B-11177 was rehydrated from *Vibrio fischeri* Reagent (Aboatox, Turku, Finland) using 2% NaCl (further referred to as 2% saline), stabilized for 1 h and used in test according to the procedures worked out by the manufacturers (BioTox™; Aboatox OY, Turku, Finland). The number of *V. fischeri* cells in the test was 1.6 ± 0.17 × 10^7^ viable cells per mL. Testing was performed 20 °C instead of 15 °C recommended by standard operational procedure of Microtox™ (AZUR Environmental, Carlsbad, CA, USA) because very few luminometers allow to adjust the temperature at lower than 20 °C level. 20 °C has been also previously used for *V. fischeri* assay [37] and for *Photobacterium phosphoreum* KCTC 2852 strain the optimal temperature (10–30 °C were compared) for bioluminescence was 20 °C [36].

### 2.3. Bioluminescence Inhibition Toxicity Assay: Testing Procedure

The kinetic bioluminescence inhibition assay (Flash-Assay) was conducted essentially as described in Mortimer *et al*. [13]. The testing with *E. coli* was performed at 30 °C and with *V. fischeri* at 20 °C. Briefly, 100 μL of the exponentially diluted test compound(s) was pipetted into 96-well polypropylene white microplates (Greiner Bio One, Germany) in duplicate. For each chemical, 5–7 sequential exponential dilutions were analysed. Into the control wells 100 μL of MilliQ water (for *E. coli*) or 2% NaCl (for *V. fischeri*) was pipetted in four replicates instead of test chemicals. 100 μL of the bacterial suspension (prepared as described above) was automatically dispensed into the wells and after that luminescence was continuously recorded during the first 30 seconds of exposure (every 0.2 s) and then once after 15 and 30 min of incubation. Fluoroskan Ascent FL plate luminometer and the Ascent Software Version 2.4.1 (both Labsystems, Helsinki, Finland) were used to guide the dispensing and measurement. Results of 2–3 independent repeats of each experiment performed in different days were used for the calculation of EC_50_ values (see below). Inhibition of the luminescence (INH%) by a certain concentration/dilution of chemical was calculated as follows:

(2) $$\mathrm{INH}\%=100-\left(\frac{IT_{t}*100}{IC_{t}}\right)$$

where:

*IT_t_*—luminescence of bacteria exposed to certain concentration of chemicals after certain time of exposure (t = 30 s, 15 min, 30 min);
*IC_t_*—luminescence of bacteria in the control solution after certain time of incubation (t = 30 s, 15 min, 30 min).

30-s, 15-min and 30-min EC_50_ values (the concentration of chemical which reduces the luminescence of bacteria by 50% after contact time of 30-s, 15-min or 30-min, respectively) were determined from concentration *versus* INH% curves. The concentration-effect curves used for the 30-s, 15-min and 30-min EC_50_ calculations were fitted with REGTOX software for Microsoft Excel™ using the log-normal model [38]. Toxicity data for different bacterial strains and/or test conditions were compared by using t-test: two-sample assuming equal variances at p < 0.05. Statistical significance of the R values of the linear regression depending on the number of data pairs was evaluated at p = 0.10, p = 0.05 and p = 0.01.

## 3. Results and Discussion

Four heavy metals and three organic compounds with different mechanisms of action were chosen to compare the performance of constitutively luminescent *E. coli* strains and against that of *V. fischeri*. As previously reported, the toxic mechanism of metals is mostly due to the interference with cofactor-metals in the active sites of enzymes [39]. All the three chosen organic chemicals are according to the Verhaar classification scheme [40] categorized as polar narcotic compounds, *i.e.*, acting non-specifically on cellular membranes. In addition, 3,5-dichlorophenol is widely used as a standard in various toxicological tests [41] and anilines are also standard chemicals in the FP6 project OSIRIS that aims to work out integrated test strategies for toxicity testing relevant for REACH [42].

### 3.1. The Effect of Exposure Time on the Toxicity of Chemicals to Luminescent Bacteria

**Figure 1.**
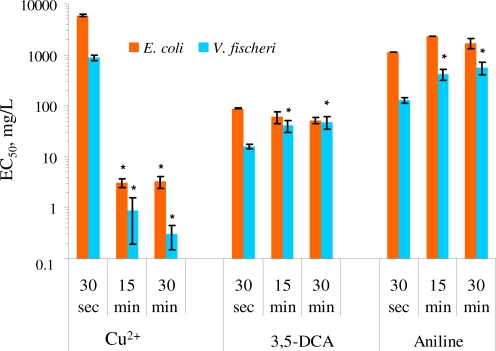
Effect of the exposure time on the toxicity (EC_50_, mg/L) of Cu^2+^ (tested as CuSO_4_), 3,5-dichloroaniline (3,5-DCA) and aniline towards luminescent recombinant *Escherichia coli* MC1061(pSLlux) in Leu-saline, 30 °C (orange bars) and *Vibrio fischeri* in 2% saline, 20 °C (blue bars). (* the EC_50_ value is different from the 30-s EC_50_ value for the same chemical and same bacterium, p < 0.05. Note the logarithmic scale of the Y-axis).

For the analysis of the effect of incubation/exposure time on the test results, the toxicity of CuSO_4_, 3,5-dichloroaniline and aniline on *E. coli* MC1061(pSLlux) in Leu-saline and *V. fischeri* in 2% saline after 30 s, 15 and 30 min of exposure was compared. As *E. coli* and *V. fischeri* are both Gram-negative bacteria with analogous cell envelope structure, their response to most of the chemicals should be theoretically comparable. Indeed, the kinetic toxicity patterns for *E. coli* and *V. fischeri* were largely similar: both anilines but not copper reduced bacterial luminescence already after 30 s of contact. The increased toxicity of copper at 15 and 30 min of incubation compared to 30 s of exposure was significantly different (p < 0.05) for both, *E. coli* and *V. fischeri* (Figure 1). In case of *V. fischeri*, the increase of the toxicity of heavy metals with increasing exposure times has been shown since long time [43–45] and 30 min exposure time has been recommended for the toxicity analysis of heavy metals with Microtox assay [46]. Figure 1 shows that the kinetics of the toxic effect of anilines for *E. coli* was different from *V. fischeri.* Namely, compared with initial inhibitory effect of anilines during first 30 s of exposure their toxicity towards *V. fischeri* remarkably (3–4 fold) decreased at 15 or 30 min of incubation (p < 0.05) and analogous “recovery” effect was not observed for *E. coli.* Thus, it could be assumed that *V. fischeri* was rapidly adapted to the (sub)toxic effect of anilines. One of the known rapid adaption mechanisms that protect the cells against the presence of toxic organic compounds in some vibrios is the fatty acid *cis-trans* isomerization reaction [46,47]. This short-term *in situ* mechanism is in terms of metabolic energy most efficient as it is post-synthesis modification process where existing lipids are used as the substrate and *de novo* lipid synthesis is not required. Given the time scale of changes in toxicity that we observed in our experiments (up to 30 min), *in situ cis-trans* isomerization of the membrane fatty acids might be the mechanism for the recovery.

### 3.2. The Effect of the Test Medium Composition on the Toxicity of Chemicals to Luminescent Bacteria

The composition of the test medium to be used for acute bioluminescence inhibition assay should be a compromise: on one hand to support the stable luminescence of the bacteria during the exposure time at sufficient level needed for the detection and on the other hand, to be as little complexing as possible. The latter is especially crucial if heavy metals are analyzed [36]. However, given luminous strains of e.g., *Bacillus, Staphylococcus, Pseudomonas* or *Escherichia* are also of medical and hygienic importance, *i.e.*, also bacterial growth inhibition data need to be studied, various nutritional supplements in the test media may be essential, depending on the auxotrophy of the strains tested. The conventional laboratory mineral growth medium for *E. coli* is M9 [32] that may need supplementation by various nutrients, depending on the strain. However, M9 basal medium contains phosphates and calcium (see Material and Methods) that may complex heavy metals and decrease their bioavailability to test bacteria. In addition, amino acids that must be supplemented to the growth/test media in case of amino acid auxotrophic bacterial strains may be responsible for additional complexation of heavy metals.

To study the effect of phosphate- and amino acids containing media on results of bacterial toxicity test, we compared the toxicity of seven standard chemicals to bioluminescent *E. coli* strains: (i) in 50% GAA-M9 medium as this medium has been used previously for these *E. coli* strains [25] and that in theory can also be used for chronic toxicity test and (ii) in 50% Leu-saline medium that is mimicking the test environment of the Microtox assay. Comparison of 30-min EC_50_ values of *E. coli* strains in these two media and the respective EC_50_ values of *V. fischeri* in 2% saline is presented in Table 2. For the given bacterial strains, the studied seven chemicals were of very different toxicity (the EC_50_ values were spanning for 4–5 orders of magnitude). However, the most toxic chemical for all bacterial strains was mercury and less toxic aniline (Table 2).

The apparent toxicity of heavy metals to *E. coli* in Leu-saline medium was significantly higher (p < 0.05) than in GAA-M9 medium. The biggest difference in toxicity was observed for zinc, which was more than three orders of magnitude less toxic if tested in GAA-M9. Over two orders of magnitude of decrease in toxicity were observed for cadmium (178–311 folds) and about 10–70 folds decrease for copper and mercury. Analogous test-medium dependent results were obtained for heavy metals Cu^2+^, Zn^2+^, Cd^2+^, and Pb^2+^ by Gellert *et al*. [48] who showed that the sensitivity of *V. fischeri* growth inhibition test was weakened by the presence of nutrient broth masking the toxicity by complexation. Analogously, in case of recombinant *E. coli* heavy metal sensor strains the reduced sensitivity of the biosensor in LB medium compared to GGM (glucose-glycerophosphate minimal medium) was observed, probably due to metal chelation or precipitation by LB medium components [49]. In case of anilines their toxicity to *E. coli* in both analysis media was not differing (p > 0.05) but 3,5-dichlorophenol in GAA-M9 medium was 6–9 fold less toxic than in Leu-saline (Table 2). The reason for the latter effect has to be further studied.

It is well documented but not so often taken into account in planning of the experiments and interpretation of the results that test medium composition has a strong impact on test results. Traditional Microtox™ test is conducted in 2% NaCl that is a good choice for testing of most of the chemicals. However, as 2% NaCl is not supporting bacterial growth, for chronic toxicity testing it must be supplemented with nutrients. Thus, the choice of the test medium should be made case-by-case taking into account the type of the test and nutritional requirements of test organisms. For example, as seen from our results, even amino-acid supplemented M9 medium could be successfully used in chronic bacterial test for organic compounds.

### 3.3. Comparative Toxicity of Chemicals to Different Constitutively Luminescent Bacteria

**Figure 2.**
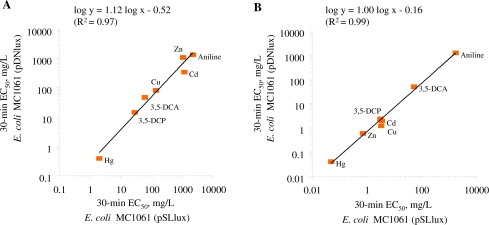
Toxicity (30-min EC_50_, mg/L) of seven standard chemicals to recombinant *Escherichia coli* strains MC1061(pDNlux) and (pSLlux) in two different media: GAA-M9 **(A)** and Leu-saline **(B)** Testing was performed at 30 °C. Log-log R^2^ value for panel A: 0.97 (p < 0.01) and for panel B: 0.99 (p < 0.01). Data are plotted from Table 2.

The two bioluminescent *E. coli* strains chosen for this study, *E. coli* MC1061(pSLlux) and MC1061(pDNlux) are otherwise identical bacteria but differ by their background bioluminescence, the former being 100-fold more luminescent than the latter. As the bioluminescence of the bacteria is metabolically costly, we assumed that due to the high energetic burden of the bioluminescence, the *E. coli* MC1061(pSLlux) strain with remarkably higher background luminescence might be more sensitive to the toxicants than *E. coli* MC1061(pDNlux). To control this hypothesis, toxicity of standard chemicals after 30 min of exposure to both *E. coli* strains in both, GAA-M9 and Leu-saline were compared (Table 2, Figure 2).

**Figure 3.**
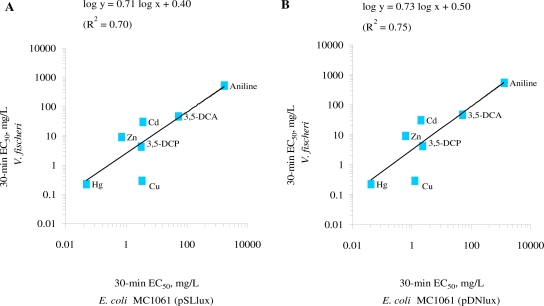
Toxicity (30-min EC_50_, mg/L) of seven standard chemicals to **(A)** recombinant *Escherichia coli* strains MC1061(pSLlux) and **(B)** (pDNLlux) in Leu-saline (30 °C) *versus Vibrio fischeri* in 2% saline (20 °C). Log-log R^2^ value for panel A: 0.70 (p < 0.05) and for panel B: 0.75 (p < 0.01). Data are plotted from Table 2.

The comparison of the toxicity of the standard chemicals (Table 2) showed that both *E. coli* strains were of comparable sensitivity in the given test medium. The log-log correlation coefficient of the 30-min EC_50_ values for these two *E. coli* strains in GAA-M9 was R^2^ = 0.97 (p < 0.01) (Figure 2(A)) and in Leu-saline R^2^ = 0.99 (p < 0.01) (Figure 2(B)). Thus, the 100-fold different background luminescence in these *E. coli* strains did not have influence on the toxicity test results showing that in the current test conditions the background luminescence was not an “energetic burden” for the cells. Analogously to our toxicity results, it has been previously shown that introducing bioluminescence to a non-luminous *Burkholderia sp*. did not affect their sensitivity towards Cu and Zn, as evaluated by dehydrogenase activity test [50]. The toxicity data obtained with both luminescent *E. coli* strains were also compared with *V. fischeri* (a Microtox™ test strain) (Table 2, Figure 3).

Although chemical-wise the EC_50_ values for most of the chemicals for *E. coli* and *V. fischeri* were statistically different (p < 0.05) except that for 3,5-dichloroaniline (Table 2), the log-log correlation coefficient of the 30-min EC_50_ values for *E. coli* (pSLlux) and *V. fischeri* was R^2^ = 0.70 (p < 0.05) (Figure 3(A)) and *E. coli* (pDNlux) and *V. fischeri* R^2^ = 0.75 (p < 0.01) (Figure 3(B)). Thus, despite of different test temperatures and slightly different composition of the test environment (2% NaCl *vs.* 0.005% Leu-supplemented 0.45% NaCl) the 30-min EC_50_ of *E. coli* and *V. fischeri* were in reasonable correlation. Indeed, Deryabin and Aleshina [35] studying the effect of content and nature of different physiological cations and anions on bioluminescence of marine bacterium *P. phosphoreum* (Microbiosensor B-17 677f) and the recombinant *E. coli* strain containing *lux* operon from *P. leiognathi* showed that factors leading to the formation of luminescent response of these two bacterial strains are universal. Therefore, given that the mechanisms of action of the test chemicals to *E. coli* and *V. fischeri* are similar, their bioluminescent response should be comparable.

## 4. Conclusions

We have shown that *Photorhabdus luminescence luxCDABE*-transformed constitutively bioluminescent bacteria are promising tools for *in vitro* toxicology in two aspects: they may replace *V. fischeri* in tests where the thermostability of the luciferase >30 °C is crucial and they can be powerful screening tools for new antimicrobials.

The kinetic Flash-Assay designed initially for toxicity analysis with naturally luminous *V. fischeri* proved suitable for artificially luminescent *E. coli.* In addition, the Flash-Assay (but not the Microtox™ test format) is applicable also for microplate luminometers and high throughput analysis. Moreover, kinetic analysis of the toxic effect of chemicals on bacterial cells may shed light on their mechanisms of toxicity and thus, facilitate the discovery of new disinfectants for clinically and hygienically important environments/surfaces, e.g., potentially contaminated with coliforms.

Last but not least, our study once more confirms the importance of addressing the composition of the test medium, which should be carefully chosen case-by-case depending on the type of the assay and nature of chemicals analyzed.

## Figures and Tables

**Table 1.** Recombinant constitutively luminescent *Escherichia coli* strains used.

***E. coli*strain**	**Description**	**Antibiotic added**	**References**
MC1061 (pDNlux)	*(araD139 Δ(ara, leu)7697 Δ lacX74 galU galK hsdR2 strA mcrA mcrB1)*^a^ *luxCDABE* genes from *Photorhabdus luminescens* in a medium-copy plasmid pDNlux ^b^	Tetracycline (10 mg/L) (Boehringer-Mannheim GmbH, Mannheim, Germany)	[25]

MC1061 (pSLlux)	Same as above but *luxCDABE* genes in a high-copy plasmid pSLlux ^c^	Ampicillin (100 mg/L) (Serva, Feinbiochemika, Heidelberg/New York)	[25]

a defective for the synthesis of leucine [33];

b Tc^r^, *luxCDABE* in pDN18N constructed in [34] were electroporated into *E. coli* MC1061 to obtain constitutively luminescent strain *E. coli* MC1061(pDNlux);

c Ap^r^, *luxCDABE* in pSL1190 constructed in [34] were electroporated into *E. coli* MC1061 to obtain constitutively luminescent strains *E. coli* MC1061(pSLlux).

**Table 2.** Toxicity (30-min EC_50_, mg/L) of seven standard chemicals to *Vibrio fischeri* and recombinant *Escherichia coli* strains MC1061(pDNlux) and (pSLlux) in different test media. The average of three independent experiments ± standard deviation is shown.

**Chemical**	***E. coli*MC1061(pDNlux)**		***E. coli*MC1061(pSLlux)**		***V. fischeri***

Test medium:	GAA-M9 ^a^	Leu-saline ^b^	GAA-M9 ^a^	Leu-saline ^b^	2% saline ^c^

Test temperature:	30 °C	30 °C	30 °C	30 °C	20 °C
Zn^2+^ (ZnSO_4_·7H_2_O)	1,089 ± 147	0.60 ± 0.083 ^d^	1,009 ± 114	0.69 ± 0.46 ^d^	9.27 ± 0.38 ^e^
Cd^2+^ (CdCl_2_·H_2_O)	345 ± 1.47	1.94 ± 1.01 ^d^	1,085 ± 441	3.49 ± 1.51 ^d^	31 ± 18.2 ^e^
Hg^2+^ (HgCl_2_)	0.39 ± 0.09	0.04 ± 0.002 ^d^	1.9 ± 0.44	0.049 ± 0.02 ^d^	0.23 ± 0.13 ^e^
Cu^2+^ (CuSO_4_)	83.5 ± 10.9	1.22 ± 0.94 ^d^	131 ± 20.1	3.26 ± 0.88 ^d^	0.30 ± 0.15 ^e^
3,5-dichlorophenol (3,5-DCP)	14.8 ± 4.04	2.29 ± 0.55 ^d^	26.9 ± 5.12	3.0 ± 0.28 ^d^	4.38 ± 0.98 ^e^
Aniline	1,362 ± 43.5	1,251 ± 39.9	2,136 ± 64.1	1,683.5 ± 388	552.4 ± 151.3 ^e^
3,5-dichloroaniline (3,5-DCA)	47.6 ± 7.34	49.8 ± 5.87	56.7 ± 8.16	51.14 ± 7.2	47.59 ± 12.8

a 50% M9 medium supplemented with 0.05% glucose and 0.25% cas-aminocids;

b 0.45% NaCl supplemented with 0.001% leucine;

c 2% NaCl;

d statistically different (p < 0.05) from the EC_50_ value in GAA-M9 for the same chemical and the same bacterial strain;

e statistically different (p < 0.05) from the EC_50_ value of the *E. coli* in Leu-saline for the same chemical.
